# Retinal Surface Macrophage Changes in Thyroid Eye Disease before and after Treatment with Teprotumumab

**DOI:** 10.1155/2022/5275309

**Published:** 2022-02-07

**Authors:** Oscar Otero-Marquez, Mona Fayad, Alexander Pinhas, Toco Y. P. Chui, Richard B. Rosen, Harsha S. Reddy

**Affiliations:** ^1^Department of Ophthalmology, New York Eye and Ear Infirmary of Mount Sinai, 310 E 14th St, New York, NY 10003, USA; ^2^Icahn School of Medicine at Mount Sinai, 1 Gustave L. Levy Pl, New York, NY 10029, USA

## Abstract

Retinal surface macrophages play key roles in the regulation of immune response, maintenance of vitreous clarity, and tissue repair. We examined the variation of parafoveal surface macrophages in a thyroid eye disease (TED) patient before and after treatment with teprotumumab (Tepezza, Horizon therapeutics). Pre- and posttreatment parafoveal surface macrophages were imaged using clinical *en face* OCT, and their density was assessed using a novel cell density mapping technique. Pretreatment, surface macrophage cell density was high. Macrophages had a nonuniform spatial distribution, and their appearance was round with few protrusions, consistent with an “activated” state. Posttreatment, cell density decreased. The macrophages were regularly spaced and had a ramified appearance and filopodia-like processes, consistent with a “quiescent” state. Surface macrophage density decreased as the Clinical Activity Score (CAS) decreased with teprotumumab treatment, suggesting a potential association of these cells with an underlying intraocular and retinal inflammatory process previously not described in TED.

## 1. Introduction

Thyroid eye disease (TED) is an autoimmune disease characterized by orbital inflammation followed by tissue remodeling and fibrosis. It is most commonly associated with Graves' disease; however, its causes are incompletely understood [1]. The insulin-like growth factor I receptor (IGF-IR), which is overexpressed by orbital fibroblasts and B and T cells in Graves' disease and TED, seems to play a key role [2]. Teprotumumab, a recently FDA-approved monoclonal antibody, targets and blocks IGF-1R and inhibits fibroblast activation via the IGF-1R/TSHR signaling complex. It has been shown to reduce proptosis, improve motility, and reduce Clinical Activity Scores (CAS) [3].

TED has been classically described as an extraocular disease; however, in some patients with active disease, intraocular findings such as optic nerve edema secondary can be seen. TED may have a component of intraocular inflammation, and evaluating intraocular biomarkers of inflammation could be a potential tool to effectively monitor the disease. One potential biomarker involves monitoring the activity of retinal surface macrophages, such as microglia and hyalocytes. These cells are the primary resident immune cells in the posterior chamber and play key roles in homeostasis and neuroprotection. They act as the first responders to neural injury and change their morphology and density in response to damage or external threats [4, 5].

Human retinal surface macrophages have previously been evaluated in healthy subjects and patients with retinopathies and glaucoma [6–8]. To the best of our knowledge, this imaging method has not yet been applied in TED. In this case report, we examine key characteristics of parafoveal surface macrophage cells in a patient with TED, before and after treatment with teprotumumab, including spatial distribution and cell morphology. In addition, we present a semiautomated cell density mapping technique that allows easy visualization and interpretation of cell distribution, with a potential for clinicians to better grade the severity of disease activity and measure treatment effect. This case was HIPAA compliant with protection of any identifiable information and adhered to the tenets of the Declaration of Helsinki.

## 2. Case Presentation

**Figure 1:**
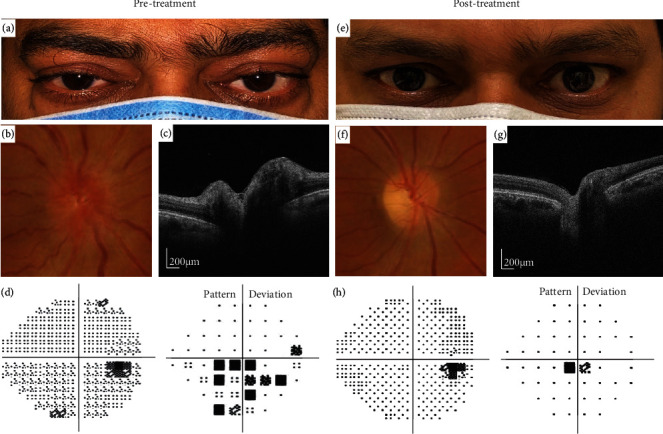
Pre- and post-teprotumumab evaluation of a patient with thyroid eye disease. (a) and (e) External photos show improvement in orbital congestion after treatment. (b) and (f) Right eye, color fundus pictures show improvement in ONH edema and less congestive vasculature. (c) and (g) Right eye, OCT-scans of the ONH show less edema after treatment. RFNL thickness decreased from 209 *μ*m to 107 *μ*m. (d) and (h) Right eye, central 24-2 Humphrey visual field (HVF). HVF prior to treatment showed central and inferior visual field defects. HVF after 5 infusions of teprotumumab with significant improvement in visual defects.

A 45-year-old male with history of Graves' disease and long-term TED on methimazole 20 mg/day and prednisolone 50 mg/day presented with worsening vision and light sensitivity. His visual acuity was 20/25 in both eyes. Physical examination revealed bilateral proptosis with Hertel's exophthalmometry readings of 24 mm (right) and 22 mm (left), eyelid edema, conjunctival injection, chemosis (Figure 1(a)), and intraocular pressures of 21 mmHg in his right eye and 20 mmHg in his left eye. His CAS was 3/7. Fundus examination revealed bilateral optic nerve head (ONH) edema (Figure 1(b)). OCT scan (DRI OCT Triton plus, Topcon Corp, Tokyo, Japan) of the posterior pole evidenced bilateral ONH edema, more severe in the right eye (Figure 1(c)). RFNL thickness was 209 *μ*m in the right eye and 137 *μ*m in the left eye. Visual field defects were identified (Figure 1(d)). In the figures, only the right eye is shown. Treatment with teprotumumab was initiated.

To assess retinal surface macrophages, ten 3 × 3 mm scans centered at the fovea were acquired (Avanti RTVue-XR; Optovue, Fremont, CA, USA) and averaged pre- and posttreatment (after the 5th infusion).

**Figure 2:**
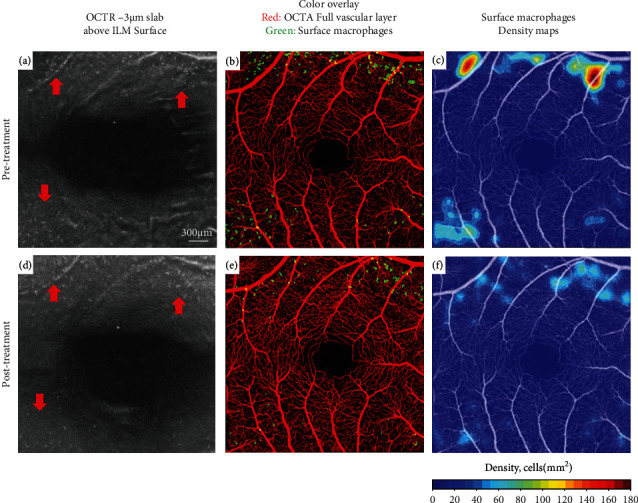
Right eye. In vivo imaging of parafoveal surface macrophages and the underlying microvasculature pre- (top row) and post-teprotumumab treatment (bottom row), using clinical OCT. (a) and (d) OCT-R slab located 3 *μ*m above the ILM surface shows the presence of macrophages. Red arrows indicate the presence of surface macrophages where significant differences in cell density and/or morphology can be seen. (b) and (e) Overlay of surface macrophages and corresponding OCT-A full vascular layer. (c) and (f) Overlay of macrophage density map and OCT-A. Surface macrophage density decreased as the inflammatory burden decreased with teprotumumab treatment.

Semiautomated macrophage identification was performed on a 3 *μ*m OCT-reflectance (OCT-R) slab located above the inner limiting membrane (ILM) surface using MATLAB (2018b; MathWorks, Natick, MA) (Figures 2(a) and 2(d)). The OCT angiography (OCT-A) full vascular slab located between the ILM and 9 *μ*m below the posterior boundary of the OPL was used for FAZ delineation and capillary density measurements of the entire OCT-A scan (Figures 2(b) and 2(e)).

**Figure 3:**
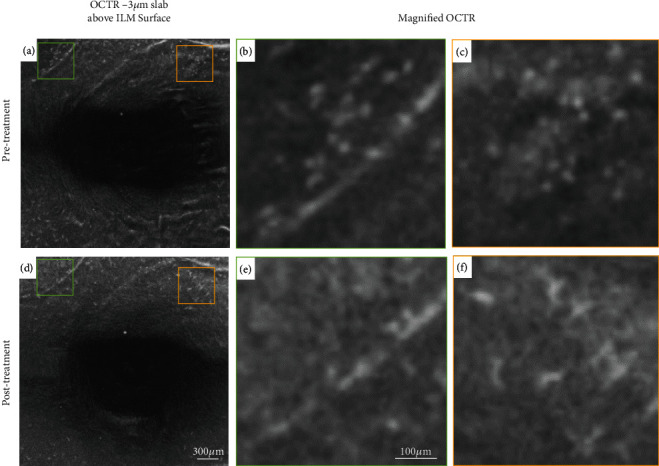
Right eye, surface macrophages distribution and morphology pre- (top row) and post-teprotumumab treatment (bottom row). (a) and (d) OCT-R slab located 3 *μ*m above the ILM surface (green and orange outline correspond to two different regions of interest). (b) and (c) Pretreatment, the cells looked plump with fewer protrusions, clustering around large vessels and areas of metabolic stress. Posttreatment, (e) fewer cells were visible and (f) appeared slender with spindle- or star-like configuration and with a uniform spatial distribution.

Surface macrophage density maps were generated in both visits to allow for easy visualization and rapid interpretation of cell distribution across the 3 × 3 mm scan area (Figures 2(c) and 2(f)). Pretreatment, surface macrophage cell density was higher. Macrophages had a nonuniform spatial distribution, and their appearance was round with few protrusions, consistent with an “activated” state (Figures 3(b) and 3(c)).

Posttreatment, orbital congestion and ONH edema improved significantly (Figures 1(e)–1(g)). The light sensitivity resolved, and his vision returned to baseline (20/20 OU). Hertel's exophthalmometry readings decreased to 21 mm (right) and 20.5 mm (left), IOPs remained stable, and his CAS improved to 0/7. RFNL thickness decreased to 107 *μ*m in the right eye and 109 *μ*m in the left eye. Visual field defects resolved as well (Figure 1(h)). Fewer surface macrophages were seen (Figure 3(e)). Cell density decreased from 9.10 to 6.55 cells/mm^2^ in the right eye and from 5.75 to 3.35 cells/mm^2^ in the left eye. The macrophages were regularly spaced and had a ramified appearance consistent with a “quiescent” state (Figure 3(f)). No significant change in capillary density was observed between the pre- and posttreatment imaging sessions. Of note, ten 4.5 mm × 4.5 mm scans centered at the ONH were also acquired pre- and posttreatment. However, surface macrophage evaluation at the initial presentation was not possible due to edema and retinal folds around the ONH which made cell identification nonviable and comparison between visits not feasible.

## 3. Discussion

In the retina, as in the rest of the CNS, microglia and related macrophage-like cells, such as hyalocytes, act as sentinels, playing key roles in homeostasis, neuroprotection, regulation of immune response, and tissue repair [4, 5]. Until recently, studies of surface macrophages were predominantly performed ex vivo or in animal models using staining and confocal imaging modalities. Nowadays, advances in noninvasive retinal imaging have enabled the visualization of retinal components at a cellular level in humans using clinical optical coherence tomography (OCT) and adaptive optics-OCT (AO-OCT) [6–8]. These investigations have visualized dynamic cells at the vitreoretinal interface with a ramified morphology and mobile soma and processes, consistent with the appearance of macrophages.

Prior research has shown that these immune cells assume a variety of conformational shapes to fulfill these functions and are broadly active in two different phenotypes, depending on whether they serve a neuroprotective or a neurotoxic role [9]. In the neurotoxic or “activated” state, microglia and related macrophage-like cell processes shorten, and their cell bodies change into an amoeboid state with plumper cell bodies, as they proliferate and migrate to the site of injury or disease, releasing inflammatory factors and becoming highly phagocytic [6]. In the neuroprotective phenotype or “quiescent” state, these cells are characterized by spindle cell bodies and ramified filopodia-like processes that constantly probe the local environment, releasing anti-inflammatory and neurotrophic factors [10].

In our patient, before treatment with teprotumumab, we observed a higher density of surface macrophage cells. In addition, these cells were round in appearance, with a nonuniform spatial distribution, and appeared to cluster around vessels and regions of metabolic stress, compatible with the neurotoxic “activated” state previously described in the literature. Posttreatment, as orbital congestion and ONH edema improved, surface macrophage cell density decreased. The distribution and appearance of parafoveal macrophages also changed with treatment. Surface macrophages became more regularly spaced and changed their morphology to a slender and ramified appearance, compatible with the neuroprotective “quiescent” state.

Our findings are consistent with the notion that TED is a systemic inflammatory disease that affects not only the periorbital tissues but the eye itself. In TED, orbital fibroblasts express IGF-1R at higher levels and secrete proinflammatory cytokines such as interleukin-1*β* (IL-1*β*), IL-1*α*, IL-6, IL-8, macrophage chemoattractant protein-1 (MCP-1), and transforming growth factor-*β* (TGF-*β*) to perpetuate orbital inflammation [11]. In addition, a recent study by Spadaro et al. [12] provides compelling evidence that macrophages in an activated state secrete IGF-1 and express IGF1-R suggesting an auto or paracrine role of this growth factor in macrophage function, and thus, a possible activation and proliferation of retinal surface macrophages in TED. Another possibility is that an ischemic etiology may be causing the intraocular inflammatory response [11, 13]. IGF-1R and TGF-*β* are well known to induce hypertrophic and proliferative responses in vascular smooth cells, thereby leading to an increase in wall thickness, decreased perfused luminal areas, potentially resulting in subclinical ischemia in TED. Although we did not find a significant change in capillary and noncapillary densities after teprotumumab treatment, minimal changes in vessel thickness after treatment could be correlated with resolution of subclinical ischemia and less activation of surface macrophages. These findings should be interpreted with caution, and further observational studies are needed to confirm whether the reduction of inflammation alone is responsible for the surface macrophages changes or mediated by teprotumumab directly.

In conclusion, clinical OCT is capable of imaging parafoveal surface macrophages in patients with TED, and is able to monitor changes in distribution and morphology and quantify changes in cell density with treatment. Our results suggest a potential association of the presence of “activated” macrophages with an underlying intraocular and retinal inflammatory process previously not described in TED. Furthermore, our results suggest the potential clinical utility in monitoring these cells to assess disease activity and response to treatment.

## Data Availability

Relevant data is available on request. Please contact Dr. Oscar Otero-Marquez (oscar.otero@mountsinai.org).
